# Effectiveness of airway clearance techniques versus control in non-hospitalized infants with moderate acute viral bronchiolitis: A randomized controlled clinical trial

**DOI:** 10.1016/j.clinsp.2025.100735

**Published:** 2025-08-04

**Authors:** Vanesa González-Bellido, Noelia Rama Suárez, Gustavo Adolfo Yánez Yepez, Maria del Carmen Jimeno Esteo, Rubén García Caraballo, Sagrario Mayoralas Alises, Juan Nicolás Cuenca Zaldívar, Márcio Vinícius Fagundes Donadio, Eleuterio A. Sánchez Romero, Samuel Fernández Carnero

**Affiliations:** a Fisiobronquial Physiotherapy Clinic, Madrid, Spain; b Pediatric Service, Hospital QuirónSalud Valle del Henares, Madrid, Spain; c Fisiobronquial Physiotherapy Clinic, A Coruña, Spain; d Emergency Service, Hospital da Barbanza, A Coruña, Spain; e Managing Director Hospital QuironSalud San José, Madrid, Spain; f Research Group in Nursing and Health Care, Puerta de Hierro Health Research Institute-Segovia de Arana (IDIPHISA), Madrid, Spain; g Universidad de Alcalá, Facultad de Enfermería y Fisioterapia, Departamento de Fisioterapia, Grupo de Investigación en Fisioterapia y Dolor, Spain; h Interdisciplinary Research Group on Musculoskeletal Disorders, Madrid, Spain; i Laboratory of Pediatric Physical Activity, Centro Infant, Pontifícia Universidade Católica do Rio Grande do Sul (PUCRS), Porto Alegre, Brazil; j Department of Physiotherapy, Facultad de Medicina y Ciencias de la Salud, Universitat Internacional de Catalunya (UIC), Barcelona, Spain; k Department of Rehabilitation Sciences, Florida Gulf Coast University, Fort Myers, USA; l Physiotherapy and Orofacial Pain Working Group, Sociedad Española de Disfunción CraneomandibularyDolor Orofacial (SEDCYDO), Madrid, Spain

**Keywords:** Airway clearance techniques, Chest physiotherapy, Autogenic drainage, Prolonged slow exhalation, Bronchiolitis, Infant, Rehabilitation

## Abstract

**Background:**

Acute viral bronchiolitis is the most common lower respiratory tract infection in infants < 2-years of age. Airway Clearance Techniques (ACTs) aim to reduce airway obstruction, thereby decreasing airway resistance, improving gas exchange, and reducing respiratory load, all of which can lead to improved clinical stability.

**Methods:**

One hundred ninety-two infants were randomly allocated to one of the following ACTs using flow-based techniques: Assisted Autogenic Drainage (AAD; *n* = 62), Prolonged Slow Expiration (PSE; *n* = 63), or a control group (*n* = 67). The sessions were performed in an outpatient setting, with a duration of 20 min and repeated 48 h later. The outcome measures were the Acute Bronchiolitis Severity Scale (ABSS), Bronchiolitis Score of Sant Joan de Déu (BROSJOD), and peripheral oxygen saturation (SpO_2_) recorded immediately after each intervention.

**Results:**

Both airway clearance techniques, PSE and AAD, demonstrated significant improvements in clinical severity scores (ABSS and BROSJOD) and SpO_2_ levels compared to the control group, with PSE showing the most pronounced effects. ABSS (ATSboot *p* = 0.001; η^2^_p_ = 0.105) and BROSJOD (ATSboot *p* = 0.003; η^2^_p_=0.037) significantly differed and interacted with time, decreasing from baseline to 48 h post-treatment. PSE outperformed the controls in reducing ABSS (*p* < 0.001; Hedges' *g* = 1.4 vs. *g* = 0.37). ACTs improved ABSS and BROSJOD scores compared to controls, with PSE showing the greatest SpO_2_ increase and wheezing reduction at 20 minutes and one hour post-baseline. At 48 h, the AAD and PSE groups had greater ABSS changes than the controls and fewer retractions at 20 min post-baseline. The AAD group had a lower heart rate, while the controls had higher respiratory rates, and the PSE group had the lowest heart rate. No significant clinical adverse effects were observed.

**Conclusions:**

Both ACTs using flow-based techniques significantly reduced ABSS and BROSJOD scores immediately after treatment and 48 h later in non-hospitalized infants with moderate acute viral bronchiolitis than in the control group. However, PSE intervention showed greater effectiveness in reducing ABSS and increasing SpO_2_ than the control and AAD group**s.**

## Highlights

• Randomized controlled trial with 4-week follow-up.
• Supervised ACTs improve short-term outcomes in infants with AVB.
• ACTs reduce clinical symptoms in viral bronchiolitis.
• Respiratory rate and retractions improved with physiotherapy.
• PSE showed greater ABSS and SpO_2_ improvements vs. AAD and control.

## Introduction

Acute Viral Bronchiolitis (AVB) is the most common lower respiratory tract infection in infants younger than 2-years.^1^ It is caused by a viral infection, most commonly Respiratory Syncytial Virus (RSV), and typically occurs in seasonal patterns, with a significant burden on infants, families, and the healthcare system.^2^ It is usually a mild to moderate disease, characterized by acute inflammation, edema, necrosis of the epithelial cells of the small airways, and increased mucus production, causing hyperinflation, wheezing, and occasionally atelectasis.^3^ However, in approximately 1 % of cases, severe disease develops and hospitalization is required.^4^ The treatment of AVB in infants is mainly symptomatic and supportive. Supplemental oxygen, fluid therapy, and respiratory support remain the mainstay of treatment. However, there is still high variability in clinical practice regarding the use of additional treatments such as Airway Clearance Techniques (ACTs) and inhalation of hypertonic saline.^5^^,^^6^ According to the latest evidence published to date, chest physiotherapy has shown a mild to moderate significant benefit in reducing the severity of acute bronchiolitis in infants, suggesting its potential utility in clinical settings for moderate AVB.^7^, ^8^, ^9^

Chest physiotherapy aims to decrease airway obstruction, reduce flow resistance, and alleviate breathing by enhancing mucociliary transportability.^7^, ^8^, ^9^, ^10^, ^11^, ^12^, ^13^ Although it is widely used in children with chronic respiratory diseases, there is contradictory evidence supporting its benefits in children with AVB.^4^^,^^5^ Techniques based on slow passive expiration, such as Prolonged Slow Expiration (PSE) and Assisted Autogenic Drainage (AAD) appear to be more effective than conventional ones.^3^ The few studies that tested these techniques (PSE and AAD) in AVB presented favorable results, suggesting that the use of appropriate ACTs of Flow-Based Techniques in the management of AVB could be considered depending on the severity of the disease.^14^, ^15^, ^16^, ^17^, ^18^, ^19^

ACTs initially focused on the impact of airflow on mucus transport and considered biomechanical and biochemical effects, supporting their use in children with acute bronchiolitis to alleviate airway obstruction.^20^^,^^21^ In addition, there is evidence that the association between slow expiratory techniques and hypertonic saline inhalation may lead to acute benefits,^22^ although the use of hypertonic saline alone remains controversial.^23^ A recent literature review has shown that even in studies where positive effects of slow expiratory techniques were not demonstrated, there were no reports of clinical worsening or destabilization of the infants, although future studies are advised to further evaluate the effects and safety of using slow expiratory techniques in non-hospitalized infants with moderate bronchiolitis.^9^

Although there is considerable evidence of the use of ACTs in infants admitted for AVB,^9^ very little is known about their use in an outpatient setting. In their 2023 Cochrane systematic review, Roqué-Figuls et al. stated the need to develop clinical trials on physiotherapy techniques for infants with moderate bronchiolitis treated in an outpatient setting, as there is currently only limited evidence.^8^

With AVB's high prevalence and morbidity, and a lack of evidence for ACTs in non-hospitalized infants, this study's main objective was to assess the effectiveness of ACTs as PSE and AADs combined with bronchodilator and hypertonic saline in non-hospitalized children with moderate AVB in improving clinical status and reducing complications of bronchiolitis.

## Materials and methods

### Trial design

This study was a three-arm, randomized controlled clinical trial conducted across two Fisiobronquial Centers in La Coruña and Madrid (Spain). The parents of all the children were fully informed and signed an informed consent form prior to participation. This study was approved by the Hospital Príncipe de Asturias Research Ethics Committee (LIB 02/2020) and was registered at clinicaltrials.gov (NCT04553822). All the procedures were performed in accordance with the principles of the Declaration of Helsinki. CONSORT (Consolidated Standards of Reporting Trials) guidelines were applied to conduct this study.^24^

### Participants

#### Eligibility and recruitment

**Fig. 1:**
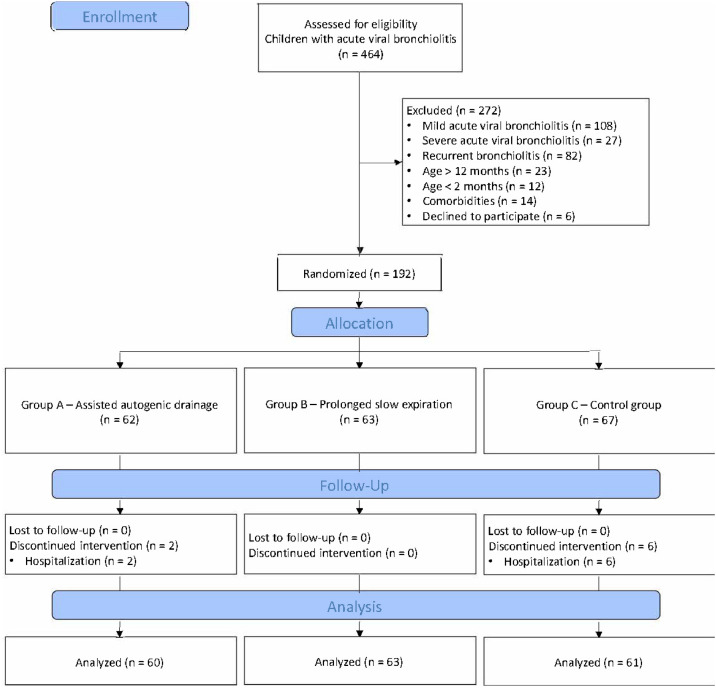
CONSORT flow chart.

Convenience sampling was performed among infants treated in the clinics where the study was conducted. After applying the eligibility criteria, 192 infants were included in this study between November 2021 and March 2022. Infants were randomized into three different groups: AAD (Group A; *n* = 62, 47 % female), PSE (Group B; *n* = 63, 40 % female), and control (CG, Group C; *n* = 67, 46 % female). Fig. 1 presents the complete flowchart of the study selection process.

Inclusion criteria

1. Age between 2 and 12 months.
2. Medical diagnosis of the first episode of AVB.
3. Moderate severity of AVB according to both the Acute Bronchiolitis Severity Scale (ABSS; ≥ 5 and ≤ 9) and the Bronchiolitis Score of Sant Joan de Déu (BROSJOD; ≥ 6 and ≤ 10).
  Peripheral oxygen saturation (SpO_2_) ≥ 94 %.
4. Informed consent provided by a legal guardian.

Exclusion criteria

1. Presence of cyanotic congenital heart disease.
2. Previous hospitalizations for recurrent wheezing or bronchospasm.
3. Presence of laryngeal conditions.
4. Non-compliance with the severity scale values and age of the patients.

Mild and severe Acute Viral Bronchiolitis (AVB) cases were excluded based on the evidence and clinical guidelines. According to the Cochrane Review by Roqué-Figuls et al. (2023), chest physiotherapy is contraindicated in patients with severe AVB because of critical respiratory status and possible comorbidities. Similarly, in mild AVB, respiratory symptoms typically decrease rapidly without intervention, with physiotherapy justified only in cases of hypersecretion. Moderate AVB represents the severity level at which chest physiotherapy demonstrates the greatest impact on reducing symptoms. Additionally, as this study was conducted in an outpatient center, the authors focused on moderate AVB cases to align with these clinical and logistical considerations.

#### Randomization and blinding

Allocation concealment was achieved using sealed opaque envelopes containing a computer-generated allocation sequence (https://www.graphpad.com/quickcalcs/randomize1/). Randomization was performed using randomly permuted blocks of sizes 6 and 9. The block sizes were alternated to maintain balance and unpredictability. The allocation sequence was stratified by study site to ensure an equal distribution of participants across the two Fisiobronquial Centers. The envelopes were prepared by a physiotherapist who was not involved in the clinical phase and were numbered sequentially. They were opened only after participant enrollment to ensure unbiased allocation. Blinding was applied to the pediatric staff and outcome evaluators. Although the outcome assessors and healthcare providers were blinded to group allocation, parents were not blinded due to the visible nature of the intervention. The same pediatrician conducted all the evaluations at T0, T20, T40, and T60. The pediatrician was responsible for administering the clinical scoring scales (ABSS and BROSJOD) and recording oxygen saturation levels, but was blinded to the type of physiotherapy intervention received by the child. Blinding was maintained by ensuring that the physiotherapists delivering the interventions did not communicate details of the treatments to the pediatrician. Physiotherapists delivering the interventions were not blinded due to the nature of the treatments but were not involved in any aspect of outcome assessment to avoid bias (Fig. 1).

### Interventions and experimental workflow

#### Clinical workflow

Patients with a clinical diagnosis were referred by their pediatrician to one of two outpatient centers for physiotherapy management. After the initial eligibility assessment, patients diagnosed with AVB who fulfilled the inclusion criteria were invited to participate in this study. Following a complete explanation of the study aims and possible associated adverse events, children whose parents or legal guardians signed an informed consent form were included in the study and forwarded to a pediatrician for clinical evaluation. The maximum time from the pediatrician’s diagnosis of Acute Viral Bronchiolitis (AVB) to the first respiratory physiotherapy session was 24 h. This ensured timely inclusion and intervention, according to the study protocol.

#### Evaluation timeline

• T0 (baseline): Evaluations by pediatricians (ABSS, BROSJOD, and SpO_2_) and administration of salbutamol (100 µg/dose).
• T10: Evaluations 10-minutes after salbutamol administration.
• T20: Administration of hypertonic saline for 8 minutes.
• T30: Evaluations 10-minutes after hypertonic saline administration.
• T40: Start of physiotherapy intervention.
• T50: Evaluations immediately post-physiotherapy.
• T60: Final evaluations.

During the 48 h break, the children did not receive any respiratory physiotherapy at the centers or elsewhere. They continued taking prescribed medications, as directed by their physician.

All treatments were performed in the presence of the parents or legal guardians. The Parent/Guardian Information Sheet, approved by the Hospital Príncipe de Asturias Research Ethics Committee, allowed parents to interrupt therapy at any point, ensuring their involvement throughout the intervention.

**Fig. 2:**
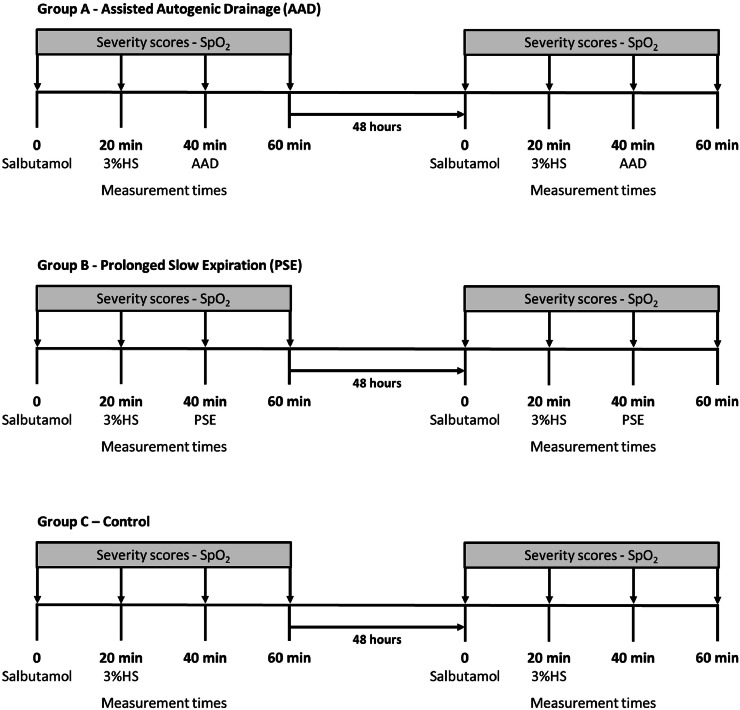
Protocol timeline for Airway Clearance Techniques (ACTs) in non-hospitalized infants.

The same timeline was followed for infants in the control group, but no physiotherapy maneuvers were performed. The protocol was implemented and performed by physiotherapists with more than 10-years of experience. The pediatricians who evaluated the patients had 10–30 years of clinical experience. The follow-up evaluation was repeated 48 h later (Fig. 2).

### Airway clearance techniques (ACTs) protocols

In all groups, treatment was initiated with a bronchodilator (salbutamol 100 µg/dose) prescribed by a pediatric physician, administered via a holding chamber (Philips® Optichamber Diamond with face mask), followed by nebulized hypertonic saline (3 %) using a vibrating mesh nebulizer (Philips Respironics, Pennsylvania, USA).^25^ Provoked coughing was induced using brief pressure applied to the trachea above the sternal notch during the inspiratory phase. This standardized maneuver facilitated secretion clearance in infants who were unable to cough effectively on their own.

• Prolonged Slow Expiration (PSE): A passive expiratory assistance technique was applied using slow thoracoabdominal pressure from the end of spontaneous inspiration until the end of expiratory volume.
• Assisted Autogenic Drainage (AAD): Based on autogenic drainage principles, manual modulation of functional breathing levels is used to optimize airflow at targeted airway generations. Gentle and progressive maneuvers were applied to stabilize the abdominal wall to avoid paradoxical movements with no excessive thoracic compression.^15^

Each session lasted 20 minutes and followed a standardized sequence: Nasal Irrigation (NI), the assigned airway clearance technique (PSE or AAD), and provoked coughing.

1. Nasal Irrigation (NI): Administered using a nasal nebulizer with 3 mL of saline per nostril while the infant was seated with their head slightly forward.
2. Prolonged Slow Expiration (PSE): Performed in eight cycles per set, alternating with provoked coughing. Three sets of cycles were performed during each session. PSE consisted of passive thoracoabdominal pressure applied during expiration, starting at the end of spontaneous inspiration and continuing until the end of the expiratory volume.
3. Assisted Autogenic Drainage (AAD): performed in 8 cycles per set, alternating with provoked coughing. Three sets of cycles were performed during each session. AAD focuses on manually modulating breathing to optimize airflow at targeted airway generations, using gentle and progressive pressure on the chest during inspiration to guide breathing.
4. Provoked Coughing: Conducted after each cycle using brief pressure on the trachea above the sternal notch during the inspiratory phase, followed by manual extraction of the phlegm with a finger inserted from the corner of the mouth.

Bronchodilator therapy (salbutamol) and hypertonic saline (3 %) were administered to all groups before the intervention. Infants in the control group did not receive PSE or AAD; however, all evaluations followed the same timeline.

For all ACTs, nasal irrigation in a sitting position was performed before maneuvers, and induced coughing followed these techniques.^26^^,^^27^

Nasal irrigation was performed manually once per session using a syringe to deliver 3 mL of solution into each nasal orifice with the child seated, head tilted slightly forward, and mouth opened. This position is safer and more practical for infants, as it facilitates natural drainage of nasal secretions, minimizes the risk of fluid entering the eustachian tubes, and ensures both comfort and control for the child and physiotherapist. Nasal irrigation was performed using nasal nebulizers to ensure controlled and effective delivery of the solution into each nasal orifice.

Infants in the control group received bronchodilator therapy and hypertonic saline inhalation but no ACTs. The evaluation timeline was identical across all groups.

### Outcome measures

The primary outcome was change in ABSS, and the secondary outcomes were the clinical variables evaluated: BROSJOD score, SpO_2,_ wheezing, crackles, respiratory effort, inspiration/exhalation ratio, respiratory frequency, and heart rate.

For each research center, a medical evaluator classified the children according to their clinical severity score and SpO_2_ using a pulse oximeter (Radical Touchscreen de Massimo®, Masimo Corporation, Irvine, CA, USA).

The ABSS is a validated scale^28^ for the Spanish population that combines the evaluation of wheezing, crackles, respiratory effort, inspiration/exhalation ratio, respiratory frequency, and heart rate. The scale has three levels of severity: mild, 0–4 points; moderate, 5–9 points; and severe, 10–13 points. The BROSJOD score combines evaluation of wheezing, crackles, chest retraction, inspiration/exhalation ratio, respiratory frequency, heart rate, and SpO_2_.^29^ This scale has three severity levels: mild (0–5), moderate (6–10), and severe (11–16).

Outcome measures were recorded at baseline (T0), 20 min (T20), 40 min (T40), and 60 min (T60) during both the first session and the 48 h session. These time points correspond to specific interventions, including salbutamol administration, hypertonic saline inhalation, and physiotherapy techniques, where applicable.

### Adverse effects

Adverse events were monitored and recorded during the entire treatment period and at 10- and 20-minutes post-treatment. If the parents requested treatment cessation, the reasons were documented. Adverse events, including transient changes such as bradycardia and desaturation, were monitored and documented. These events resolved spontaneously without requiring any medical intervention.

### Sample size calculation and statistical analysis

Due to the absence of previous studies with comparable methodologies and main outcome variables, the study itself was used as a pilot to calculate the final sample size. Moreover, no validated Minimal Clinically Important Differences (MCIDs) have been established for ABSS or BROSJOD scores in infants with bronchiolitis, which further limited the feasibility of conducting a conventional a priori sample size calculation. Using data from the first 20 children recruited in each group on the ABSS score, the results indicate that accepting a risk α of 0.05, with a power greater than 95 % for an estimated effect size of ϵ^2^=0.357 at 20 minutes after 48 h, 55 children per group were needed, with a total of 165 children, estimating a 25 % loss because the children were younger than 1-year and it was very easy for them not to attend the 48 h follow-up.

For statistical analysis, R Ver. 4.1.3 program was used. Software (*R* Foundation for Statistical Computing, Institute for Statistics and Mathematics, Welthandelsplatz 1, 1020 Vienna, Austria). The level of significance was set at *p* < 0.05. The Kolmogorov-Smirnov test with Lilliefors correction was used to determine the distribution of quantitative variables. Qualitative variables were described as absolute values and relative frequencies, whereas quantitative variables were described as means and standard deviations. Quantitative outcome variables were analyzed using a robust repeated measures model with two factors, between (groups) and within (measurements), due to the non-normal distribution of the variables, which was based on the use of bootstrap-based resampling techniques to calculate a pseudo-statistic bootstrapped (ATS_boot_) estimator with its level of significance that allowed us to ignore the absence of normality;^30^ for the post-hoc tests, the Mann-Whitney *U* test was applied for the interaction group, with Bonferroni correction. In addition, percentage changes after treatment were calculated using the standard formula: [(finalmeasurement−initialmeasurement)/initialmeasurement]×100.

Qualitative variables were analyzed using the Cochran (Mantel) Haenszel test after fulfilling the assumption of homogeneity of the odds ratio using the Wolf test, whereas for the post hoc tests, Fisher’s exact test was applied with Bonferroni correction. All analyses were conducted using a modified Intention-To-Treat (mITT) approach, including only participants who received the assigned intervention and completed both baseline and immediate post-treatment assessments. No imputation methods were applied for missing data.

The effect size in the quantitative variables was calculated using the statistic η^2^_p_ obtained by bootstrapping because of the non-normal distribution of the variables (bootstrapped partial eta squared), defined as small (0.01‒0.06), moderate (0.06‒0.14) and large (> 0.14). Qualitative variables were calculated using Cramer’s V and defined as small (0.058‒0.173), medium (0.173‒0.289) and large (> 0.289).

## Results

A final sample of 192 patients (44 % female) with a mean age of 6.82±2.89 months was recruited. Four participants (2.1 %) did not complete the 48 h reassessment and were excluded from longitudinal comparisons but included in baseline analyses. No further attrition occurred. There were no significant baseline differences in the clinical, demographic, or primary and secondary outcome variables between the groups; therefore, no baseline adjustments were performed (see Table 1 for detailed information).

**Table 1:** Baseline clinical and demographic characteristics of participants.

		**Assisted autogenic drainage group**	**Slow expiration group**	**Control group**	**p-value**^a^
**n**		62	63	67	
**Age (months)**		6.79±2.82	6.84±2.76	6.82±3.12	0.99
**Center, n (%)**	Galicia	14 (22.6)	18 (28.6)	11 (16.4)	0.25
	Madrid	48 (77.4)	45 (71.4)	56 (83.6)	
**Siblings enrolled in the study, n (%)**	Both	3 (4.8)	5 (7.9)	3 (4.5)	0.6
	No	17 (27.4)	23 (36.5)	19 (28.4)	
	Yes	42 (67.7)	35 (55.6)	45 (67.2)	
**Kindergarten, n (%)**	Yes	40 (64.5)	39 (61.9)	36 (53.7)	0.42
	No	22 (35.5)	24 (38.1)	31 (46.3)	
**Medication, n (%)**	Nothing	22 (35.5)	18 (28.6)	28 (41.8)	0.26
	Prednidsone	0 (0)	0 (0)	1 (1.5)	
	Salbutamol	23 (37.1)	29 (46.0)	23 (34.3)	
	Salbutamol +Antibiotic	1 (1.6)	2 (3.2)	3 (4.5)	
	Salbutamol +Budesonide	2 (3.2)	2 (3.2)	1 (1.5)	
	Salbutamol +Prednisone	12 (19.4)	9 (14.3)	11 (16.4)	
	Salbutamol + Prednisone + Antibiotic	2 (3.2)	0 (0.0)	0 (0.0)	
	Salbutamol + Prednisone + Budesonide	0 (0.0)	3 (4.8)	0 (0.0)	
**Paternal history of asthma, n (%)**	No	40 (64.5)	45 (71.4)	48 (71.6)	0.35
	Yes (both)	3 (4.8)	4 (6.3)	1 (1.5)	
	Yes (father)	12 (19.4)	12 (19.0)	13 (19.4)	
	Yes (mother)	7 (11.3)	2 (3.2)	5 (7.5)	
**SpO_2_ (%)**		95.35±0.48	95.54±0.5	95.51±0.59	
**ABSS score**		5.23±0.42	5.02±0.13	5.04±0.21	
**BROSJOD score**		6.16±0.45	6.16±0.45	6.15±0.5	

Data are expressed as the mean ± standard deviation or absolute and relative values ( %).

BROSJOD, Bronchiolitis Score of Sant Joan de Déu; ABSS, Acute Bronchiolitis Severity Scale.

a Significant if *p* < 0.05.

While transient bradycardia and desaturation were observed in some cases, these events resolved spontaneously and did not necessitate any additional medical intervention, confirming the safety of the interventions. No clinically significant adverse events were observed during the follow-up period.

**Fig. 3:**
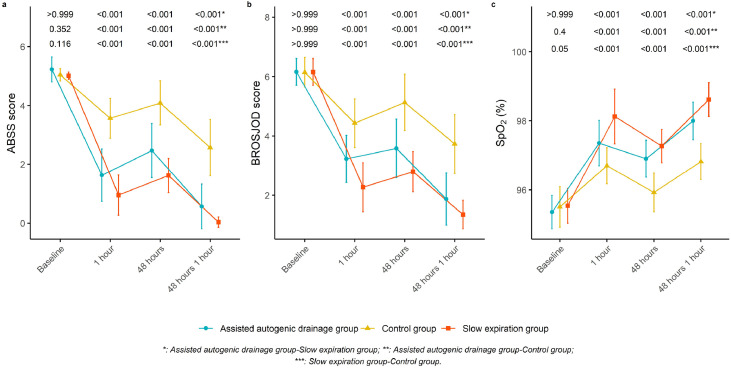
Clinical outcomes by group over time: (a) Acute Bronchiolitis Severity Scale (ABSS); (b) Bronchiolitis Score of Sant Joan de Déu (BROSJOD); (c) Peripheral Oxygen Saturation (SpO_2_).

When ABSS and BROSJOD were analyzed, the results of the repeated-measures model revealed significant differences between the groups. Time interaction was verified in the ABSS variables (ATS_boot_ *p* = 0.001) with a moderate and significant effect size (η^2^_p_ = 0.105 (0.232, 0.304)) and in the BROSJOD variable (ATS_boot_ *p* = 0.003) with a small and significant effect size (η^2^_p_ = 0.037 (0.167, 0.234). It was verified that both in the baseline measurement and after 48 h, there was a strong decrease in the scores of both scales in both physiotherapy techniques (Fig. 3).

Changes in post-treatment ABSS showed a reduction in both treatment groups compared to the control group (*p* < 0.001; Hedges’ *g* = 0.37) but were greater in the PSE group (*p* < 0.001, Hedges’ *g* = 1.4). However, in the control group, the ABSS did not change significantly immediately after treatment (*p* = 0.87; Hedges’ *g* = 0.16). The differences between the groups after the intervention were significant with a moderate effect size (*p* = 0.01; Hedges’ *g* = 0.54). Both ACTs had a significant effect on ABSS and BROSJOD compared with the control group. Regarding SpO_2_, the results obtained showed that it increased during all sessions in the three groups studied, with the increase being more pronounced after one hour from the baseline. The PSE group showed a greater increase in SpO_2_ than did the AAD group (Supplementary Material Tables 1 and 2).

However, regarding the percentage of change calculated (Table 2), it was observed that the percentage of change was higher in both ACT's than in the control group. A higher percentage change in measurements 48 h after baseline for ABSS was observed in the AAD and PSE groups. The percentage change remained similar in the AAD and control groups for BROSJOD and was somewhat lower in the PSE group. Regarding the percentage change in SpO_2_, the results showed a higher percentage change after one hour from baseline than after 48 h. This percentage change was also higher in the physiotherapy treatment groups and was specifically higher in the PSE treatment group. This prolonged effect highlights the potential of these physiotherapy techniques to offer lasting improvements in clinical stability.

**Table 2:** Change percentage 1 h ‒ baseline at treatment and 48 h posttreatment.

	**Assisted autogenic drainage group**	**Slow expiration group**	**Control group**
**Acute Bronchiolitis Severity Scale**			
Baseline ‒ 1 h	−68.6 %	−81.1 %	−29.4 %
48 h baseline ‒ 1 h	−78.8 %	−98.9 %	−36.7 %
**Bronchiolitis Score of Sant Joan de Déu**			
Baseline ‒ 1 h	−47.5 %	−63.2 %	−27.8 %
48 h baseline ‒ 1 h	−46.7 %	−51.1 %	−27.1 %
**Peripheral Oxygen Saturation**			
Baseline ‒ 1 h	2.1 %	2.7 %	1.3 %
48 h baseline ‒ 1 h	1.1 %	1.4 %	0.9 %

For the other secondary outcomes, significant differences (*p* < 0.05) in the group × time interaction were demonstrated for all quantitative variables (heart rate, beats/min; respiratory rate, breath/min), number of wheezing episodes, number of wheezing episodes with crackling, retractions, number of cracklings, inspiration-expiration rate, and number of air entrances) with significant effect sizes (Supplementary File Table 1). Supplementary File Table 3 shows the change in every secondary outcome (wheezing, crackles, respiratory effort, inspiration/exhalation ratio, respiratory frequency, and heart rate) after the intervention and the differences between the groups. As can be appreciated, all of them showed a significantly greater improvement in the experimental groups. Based on the sub-scores, wheezing improved significantly after 20 min from baseline and after one hour in the PSE group compared with the AAD and control groups. Retractions were significantly lower in both intervention groups 20 minutes after baseline than in the control group. The heart rate decreased in the AAD group compared to the control group, and the respiratory rate also decreased with the sessions and was higher in the control group than in the PSE group, as well as in the AAD group than in the PSE group (Supplementary Material Tables 3 and 4). Heart rate changes were significant across groups, showing reductions in the intervention groups compared to the control group (Supplementary Fig. 1).

Post-hoc tests and significant differences for the group × time interaction of secondary outcomes and qualitative variables are detailed in the Supplementary Material.

No significant clinical adverse effects were observed. The main adverse events were bronchial constriction (bradycardia and desaturation), chest retraction, wheezing, epistasis, vomiting, and tachycardia. Infants were classified as presenting with tachycardia whenever a heart rate >160 bpm was identified.^7^^,^^17^^,^^18^

## Discussion

The present study is the first randomized controlled clinical trial with a large sample size that evaluated the efficacy of airway clearance techniques on an outpatient basis in 192 children with acute viral bronchiolitis. Eight children were hospitalized because of worsening clinical conditions, namely respiratory failure, indicating progression from moderate to severe acute bronchiolitis. Ultimately, 184 children (44 % female) completed the study.

The findings of this study demonstrate the significant impact of ACTs on the clinical outcomes of infants with acute viral bronchiolitis. Both PSE and AAD methods showed substantial and sustained improvements in clinical parameters such as ABSS and BROSJOD, as well as SpO_2_ levels, up to 48 h post-treatment. This highlights the potential role of these techniques in accelerating recovery and reducing symptom severity, aligning with the growing need for evidence-based, non-invasive interventions in pediatric respiratory care. These results are particularly significant as they suggest not only immediate benefits following physiotherapy sessions but also a lasting effect that extends beyond the treatment window. Such prolonged improvements are critical for reducing the burden on healthcare systems and enhancing patient recovery trajectories.

Given that AVB is a self-limited disease, and its total duration is 10‒15 days with gradual resolution from 5‒7 days, the intervention of the present study was developed during that time in which the child presented symptoms (days from symptom onset 4.58±1.11 ADD; 4.57±1.19 PSE; 4.22±1.25 CG).

This clinical trial examined the efficacy of two ACTs in nonhospitalized infants aged <12-months with moderate AVB. The PSE group showed the most significant decrease in ABSS and highest increase in SpO_2_, indicating its potential for treatment. The improvements in the ABSS and BROSJOD scores were likely due to reduced wheezing, respiratory effort, and rates.

The observed differences in the ABSS and BROSJOD scales (≤2-points) occurred during a period of spontaneous recovery, raising questions about their clinical significance. Although an established Minimal Clinically Important Difference (MCID) for these scales is not available, the reductions observed in the ABSS are supported by its validated use for assessing severity. A cutoff of 10-points was identified as optimal for distinguishing patients at high risk of mechanical ventilation or PICU admission (ROC AUC=0.94). Similarly, the BROSJOD scale has been validated for severity classification, but lacks a defined MCID.^31^^,^^32^ While these changes may appear quantitatively modest, they were accompanied by significant physiological improvements (e.g., SpO_2_) and large effect sizes, and may contribute to preventing disease progression and hospitalization in clinical practice.

Despite the lack of a formal MCID, the reductions observed in ABSS and BROSJOD aligned with moderate-to-large effect sizes, suggesting meaningful clinical changes. These reductions were further supported by improvements in the secondary outcomes, including reduced wheezing, respiratory effort, and increased oxygen saturation. These results highlight the importance of symptom resolution and patient comfort as markers of therapeutic efficacy. Given that no Minimal Clinically Important Differences (MCIDs) have been formally validated for the ABSS and BROSJOD scales in this population, the authors relied on the interpretation of moderate-to-large effect sizes as indicators of potentially meaningful clinical improvement, consistent with current recommendations for non-pharmacological trials.

The immediate reduction in ABSS and BROSJOD scores observed after 20 min can be attributed to the combined effects of bronchodilator therapy and hypertonic saline, which act synergistically to reduce bronchial edema, alleviate inflammation, and enhance mucus clearance. These effects likely complement the benefits of airway clearance techniques, resulting in rapid relief of symptoms.

It is already known that conventional ACTs do not affect bronchospasm or edema associated with AVB.^16^ Disease severity may affect the effectiveness of the ACT. Studies have shown that ACTs do not worsen the clinical status of hospitalized patients with AVB.^5^^,^^14^^,^^22^^,^^33^, ^34^, ^35^ Recent studies have suggested a sustained reduction in the clinical scores of ACTs.^15^^,^^16^ Mechanical devices and manual techniques have yielded similar positive results.^17^ ACTs combined with bronchodilators and hypertonic saline are more effective, particularly for wheezing.^36^

Pinto et al.^19^ demonstrated a significant improvement in the Kristjansson Respiratory Score in the PSE intervention group compared with the control group. Conesa-Segura et al.^16^ showed that the PSE intervention group had a significantly lower ABSS score than controls, with notable reductions observed shortly after the intervention and on the last day before discharge. In comparison, the present study indicated considerable improvements in the ABSS and BROSJOD scales in children receiving PSE, with significant percentages observed after the first intervention, suggesting a rapid and substantial response to treatment.

The present results suggest that chest physiotherapy with PSE and AAD effectively cleared airway secretions, reduced bronchial obstruction, and improved the respiratory status in children with moderate bronchiolitis. Even so, the inhalation of hypertonic saline in infants with AVB remains controversial,^37^ and its use in both outpatient settings[25] and in combination with ACTs should be further studied.

The immediate improvements observed in the ABSS and BROSJOD scores after 20-min of treatment are consistent with previous studies demonstrating the efficacy of combined airway clearance techniques, bronchodilators, and hypertonic saline. Induced coughing, a widely accepted technique in pediatric respiratory physiotherapy, is particularly useful in infants who are unable to cough effectively on their own. While this technique introduces variability, efforts have been made to standardize its application by experienced physiotherapists and validated clinical scoring systems to minimize bias. These findings highlight the potential of such interventions to rapidly alleviate symptoms, although further research is required to assess their long-term impact.

### Limitations and strengths

The main limitations of the present study include the evaluation of three combined treatments (ACTs, bronchodilators, and hypertonic saline), which does not allow verification of the effect of each isolated intervention. Thus, the individual effects of ACTs must be addressed. However, the authors believe that the use of a combined protocol reflects the clinical use of such treatments more accurately.

Consequently, the clinical effects reported in this study should be interpreted as resulting from a combined therapeutic approach, rather than being attributed solely to the airway clearance techniques. An additional limitation of this study is that the patients were included between days 4.2 and 4.8 after symptom onset, potentially missing the early window of efficacy in reducing hospital admissions. Nevertheless, this timing may align with the mucus-dominant phase of bronchiolitis, when airway clearance techniques are most relevant. In the early days of the disease, airway obstruction is primarily due to inflammation and bronchospasm, whereas by days 3 to 5, mucus production becomes more prominent. Therefore, the selected timing may in fact optimize the effectiveness of ACTs. That said, the most important clinical implication of this study is that although AVB is an acute disease that usually resolves within a few days, the use of ACTs may accelerate the recovery of children and reduce the risk of hospital admission, with no significant clinical adverse effects reported. These findings provide strong evidence that airway clearance techniques (AAD and PSE) not only improve clinical parameters immediately post-intervention but also sustain these improvements over 48 h. This prolonged effect is particularly significant as it demonstrates the enduring benefits of physiotherapy, which may enhance recovery trajectories and reduce symptom burden in non-hospitalized children with acute viral bronchiolitis. Nevertheless, since the study was conducted exclusively in Spanish outpatient clinics, the findings may not be fully generalizable to other healthcare systems, particularly in low-resource settings. Future multicenter trials across diverse healthcare environments are warranted to confirm external validity.

In addition, infants with recurrent wheezing or significant comorbidities were excluded to preserve sample homogeneity. As a result, the findings may not extend to higher-risk pediatric populations, and further studies are needed to evaluate ACTs in more clinically complex cases.

To our knowledge, this study adds valuable evidence supporting the implementation of these techniques to achieve both short- and longer-term clinical benefits.

The study design, incorporating a randomized controlled clinical trial framework, addresses the gap in pediatric respiratory care by quantitatively assessing the impact of specific ACTs on acute viral bronchiolitis, thereby contributing to evidence-based practice and guiding future clinical protocols.

Additionally, the authors acknowledge that the sample size estimation was based on post-hoc analysis of pilot data, given the lack of established MCIDs for the primary outcomes. Although this approach is not conventional, it was necessary to accommodate the absence of validated clinical thresholds in the literature.

Finally, the authors believe that the present study has sound methodological quality. Given that a three-arm randomized controlled clinical trial was presented, the parents of the patients were unaware of the allocation (and because of their young age, so were the patients), as were the evaluators.

In future trials, the use of ethically appropriate sham interventions could be considered to mitigate performance bias; however, implementing such procedures in symptomatic infants poses important ethical and practical challenges.

## Conclusions

This study showed the effectiveness of Assisted Autogenic Drainage (AAD) and Prolonged Slow Expiration (PSE) combined with a bronchodilator and hypertonic saline in non-hospitalized children under 12-months of age who presented with a first episode of moderate acute viral bronchiolitis.

The PSE intervention was more effective in reducing the Acute Bronchiolitis Severity Scale (ABSS) score and increasing SpO_2_ compared to both the control and AAD groups, highlighting its significant impact on improving post-treatment respiratory symptoms.

Both ACTs of Flow-Based Techniques significantly reduced the clinical scores (ABSS and BROSJOD) and some respiratory symptoms of bronchial obstruction when compared to no ACTs of Flow-Based Techniques.

This study highlights the effectiveness of physiotherapy techniques in improving clinical parameters in infants with acute viral bronchiolitis, with both immediate and sustained benefits observed over 48 h. These findings underscore the value of implementing AAD and PSE as part of early interventions for this condition, with no clinically important adverse events identified in the follow-up period.

**Supplementary materials**: The following supporting information can be downloaded at: w ww.xxxxxx, Table S1: Quantitative outcome variables; Table S2: Qualitative outcome variables; Table S3: Quantitative outcome variables pairwise comparison; Table S4: Qualitative outcome variables pairwise comparison.

## Institutional review board statement

The study was conducted in accordance with the Declaration of Helsinki, approved by the Hospital Príncipe de Asturias Research Ethics Committee (LIB 02/2020) and registered in clinicaltrials.gov (NCT04553822).

## Informed consent statement

Informed consent was obtained from all subjects involved in the study. Written informed consent has been obtained from the patients to publish this paper.

## Data availability statement

Anonymized Individual Participant Data (IPD) and the *R* statistical code used for analysis are provided as supplementary material with this submission. Additional information can be made available upon reasonable request from the corresponding author.

## Authors’ contributions

Vanesa González-Bellido (VGB) and Samuel Fernández Carnero (SFC) had primary responsibility for supervision and project administration. Verónica Velaz-Baza (VVB), Maria del Carmen Jimeno Esteo (CJE) and Noelia Rama Suárez (NRS), had primary responsibility for investigation and writing-review & editing. Vanesa González-Bellido, Eleuterio A. Sánchez Romero (EASR) and Samuel Fernández Carnero had primary responsibility on conceptualization and methodology. Sagrario Mayoralas Alises (SMA) had primary responsibility for validation of data outcomes and contributed to the writing of the original draft. Juan Nicolás Cuenca Zaldívar (JNC) performed the formal analysis and data curation, developed de software (analytical code) and writing of theoriginal draft. Vanesa González-Bellido and Verónica Velaz-Baza contributed to the writing-original draft. Márcio Vinícius Fagundes Donadio (MVFD) and Eleuterio A. Sánchez Romero (EASR) contributed to conceptualization, writing-original draft, and writing-review & editing.

## Funding

MVFD was financed in part by Coordenação de Aperfeiçoamento de Pessoal de Nível Superior – Brasil (CAPES) – Finance Code 001, and Conselho Nacional de Desenvolvimento Científico e Tecnológico (CNPq).

## Declaration of competing interest

The authors declare no commercial or financial conflicts of interest. Some authors are affiliated with the participating clinical sites; however, their roles were limited to the delivery of interventions under standardized protocols. They did not participate in the design, data analysis, or interpretation of results. No financial incentives were involved.

## Appendix Supplementary materials

Image, application 1
