# Supplementary material for: Effectiveness of airway clearance techniques versus control in non-hospitalized infants with moderate acute viral bronchiolitis: A randomized controlled clinical trial
Source: Clinics (Sao Paulo). 2025 Aug 4;80:100735. doi: 10.1016/j.clinsp.2025.100735 (PMC12374053; doi:10.1016/j.clinsp.2025.100735)

**CLINICS-D-24-00865_Supplementary Material**

**Table S1** Quantitative outcome variables.

|  | **Assisted autogenic drainage group** | | | | | | | | **Slow expiration group** | | | | | | | | **Control group** | | | | | | | |  | | | |
| --- | --- | --- | --- | --- | --- | --- | --- | --- | --- | --- | --- | --- | --- | --- | --- | --- | --- | --- | --- | --- | --- | --- | --- | --- | --- | --- | --- | --- |
|  | **Baseline** | **10 minutes** | **20 minutes** | **1 hour** | **48 hours baseline** | **48 hours 10 minutes** | **48 hours 20 minutes** | **48 hours 1 hour** | **Baseline** | **10 minutes** | **20 minutes** | **1 hour** | **48 hours baseline** | **48 hours 10 minutes** | **48 hours 20 minutes** | **48 hours 1 hour** | **Baseline** | **10 minutes** | **20 minutes** | **1 hour** | **48 hours baseline** | **48 hours 10 minutes** | **48 hours 20 minutes** | **48 hours 1 hour** | **^a^p-value time** | **^a^p-value group** | **^a^p-value group: time** | η^2^ **(95% CI)** |
| **ABSS score** | 5.23 ± 0.42 | 4.47 ± 0.78 | 3.34 ± 0.99 | 1.63 ± 0.89 | 2.47 ± 0.92 | 2.02 ± 0.84 | 1.68 ± 0.7 | 0.56 ± 0.76 | 5.02 ± 0.13 | 4.27 ± 0.7 | 3.02 ± 0.96 | 0.95 ± 0.68 | 1.62 ± 0.58 | 1.56 ± 0.53 | 1.32 ± 0.47 | 0.03 ± 0.18 | 5.04 ± 0.21 | 4.75 ± 0.5 | 3.99 ± 0.64 | 3.57 ± 0.68 | 4.09 ± 0.75 | 3.79 ± 0.83 | 2.97 ± 1.03 | 2.57 ± 0.96 | <0.001 | <0.001 | 0.001 | 0.105 (0.232, 0.304) |
| **BROSJOD score** | 6.16 ± 0.45 | 5.37 ± 0.77 | 4.44 ± 0.76 | 3.23 ± 0.8 | 3.58 ± 0.98 | 3.18 ± 0.93 | 2.89 ± 0.58 | 1.87 ± 0.88 | 6.16 ± 0.45 | 5.46 ± 0.71 | 3.84 ± 1.08 | 2.27 ± 0.83 | 2.79 ± 0.68 | 2.73 ± 0.6 | 2.57 ± 0.56 | 1.35 ± 0.48 | 6.15 ± 0.5 | 5.91 ± 0.57 | 5.07 ± 0.8 | 4.43 ± 0.82 | 5.13 ± 0.95 | 4.85 ± 0.93 | 4.28 ± 1.13 | 3.73 ± 0.99 | <0.001 | <0.001 | 0.003 | 0.037 (0.167, 0.234) |
| **SpO_2_(%)** | 95.35 ± 0.48 | 96.03 ± 0.44 | 96.73 ± 0.75 | 97.35 ± 0.66 | 96.9 ± 0.53 | 97.26 ± 0.68 | 97.85 ± 0.6 | 98 ± 0.54 | 95.54 ± 0.5 | 96.35 ± 0.68 | 97.06 ± 0.56 | 98.13 ± 0.79 | 97.27 ± 0.48 | 97.67 ± 0.51 | 98.19 ± 0.47 | 98.62 ± 0.49 | 95.51 ± 0.59 | 95.84 ± 0.57 | 96.52 ± 0.59 | 96.7 ± 0.52 | 95.93 ± 0.56 | 96.33 ± 0.7 | 96.69 ± 0.58 | 96.82 ± 0.52 | <0.001 | <0.001 | 0.001 | 0.015 (0.144, 0.211) |
| **Hearth rate (beats/min)** | 145.06 ± 5.99 | 146.58 ± 5.48 | 141.55 ± 4.83 | 137.24 ± 5.33 | 139.11 ± 4.44 | 140.31 ± 4.14 | 137.82 ± 3.85 | 134.4 ± 3.41 | 149.67 ± 4.29 | 151.3 ± 3.61 | 146.57 ± 4.45 | 139.14 ± 4.27 | 141.17 ± 3.81 | 141.87 ± 4.42 | 139.13 ± 4.24 | 135.27 ± 3.04 | 147.43 ± 6.49 | 148.84 ± 5.9 | 145.18 ± 5.36 | 141.96 ± 3.18 | 143.72 ± 5.34 | 145.03 ± 4.89 | 142.58 ± 5.54 | 140.36 ± 4.32 | <0.001 | <0.001 | 0.009 | 0.094 (0.047, 0.094) |
| **Respiratory rate (breath/min)** | 39 ± 3.83 | 38.69 ± 3.96 | 36.87 ± 3.94 | 34.39 ± 3.65 | 35.4 ± 4.24 | 35.08 ± 4.44 | 33.74 ± 4.19 | 31.29 ± 3.53 | 38.33 ± 4.85 | 37.78 ± 5.12 | 36.06 ± 5.19 | 33.62 ± 5.29 | 33.57 ± 5.05 | 33.6 ± 5.12 | 32.52 ± 5.41 | 30.84 ± 4.48 | 37.31 ± 4.88 | 37.21 ± 5.36 | 35.81 ± 5.23 | 34.7 ± 4.97 | 36.09 ± 4.8 | 36.12 ± 5 | 34.94 ± 5.1 | 34.3 ± 5.11 | 0.237 | <0.001 | <0.001 | 0.128 (0.019, 0.054) |
| **Number of wheezing** | 1.58 ± 1.3 | 0.37 ± 0.68 | 0.1 ± 0.3 | 0.03 ± 0.18 | 0.03 ± 0.18 | 0.03 ± 0.18 | 0.03 ± 0.18 | 0.03 ± 0.18 | 1.05 ± 0.99 | 0.33 ± 0.67 | 0.1 ± 0.3 | 0 ± 0 | 0 ± 0 | 0 ± 0 | 0 ± 0 | 0 ± 0 | 0.69 ± 1.05 | 0.3 ± 0.74 | 0.09 ± 0.42 | 0.07 ± 0.36 | 0.21 ± 0.69 | 0.13 ± 0.55 | 0.09 ± 0.42 | 0.07 ± 0.36 | 0.282 | <0.001 | 0.003 | 0.096 (0.027, 0.119) |
| **Number of wheezing with crackling** | 1.76 ± 0.43 | 1.21 ± 0.41 | 1.15 ± 0.36 | 0.87 ± 0.42 | 1.05 ± 0.22 | 1.03 ± 0.18 | 1.03 ± 0.18 | 0.39 ± 0.55 | 1.35 ± 0.48 | 1.1 ± 0.3 | 1.06 ± 0.25 | 0.57 ± 0.5 | 1.02 ± 0.13 | 1 ± 0 | 1 ± 0 | 0 ± 0 | 1.51 ± 0.5 | 1.31 ± 0.47 | 1.1 ± 0.31 | 1.1 ± 0.31 | 1.13 ± 0.34 | 1.07 ± 0.26 | 1.07 ± 0.26 | 1.07 ± 0.26 | <0.001 | <0.001 | <0.001 | 0.015 (0.145, 0.215) |
| **Retraction effort** | 1 ± 0 | 0.97 ± 0.18 | 0.82 ± 0.39 | 0.37 ± 0.49 | 0.53 ± 0.5 | 0.23 ± 0.42 | 0.05 ± 0.22 | 0.03 ± 0.18 | 1 ± 0 | 1 ± 0 | 0.44 ± 0.5 | 0.05 ± 0.21 | 0.06 ± 0.25 | 0.02 ± 0.13 | 0 ± 0 | 0 ± 0 | 1 ± 0 | 0.99 ± 0.12 | 0.97 ± 0.17 | 0.9 ± 0.31 | 1 ± 0 | 0.97 ± 0.17 | 0.84 ± 0.37 | 0.7 ± 0.46 | <0.001 | <0.001 | <0.001 | 0.133 (0.248, 0.344) |
| **Number of crackling** | 1.1 ± 0.94 | 1.63 ± 0.68 | 1.71 ± 0.49 | 1 ± 0.48 | 1.53 ± 0.5 | 1.42 ± 0.5 | 1.4 ± 0.49 | 0.5 ± 0.67 | 1.06 ± 0.98 | 1.49 ± 0.67 | 1.49 ± 0.5 | 0.63 ± 0.49 | 1.03 ± 0.18 | 1 ± 0 | 1 ± 0 | 0 ± 0 | 1.33 ± 0.89 | 1.46 ± 0.7 | 1.3 ± 0.49 | 1.3 ± 0.52 | 1.24 ± 0.58 | 1.24 ± 0.5 | 1.15 ± 0.4 | 1.16 ± 0.45 | <0.001 | <0.001 | <0.001 | 0.055 (0.08, 0.137) |
| **Inspiration-expiration rate** | 1 ± 0 | 0.82 ± 0.39 | 0.4 ± 0.49 | 0.05 ± 0.22 | 0.08 ± 0.27 | 0.05 ± 0.22 | 0 ± 0 | 0 ± 0 | 1 ± 0 | 0.56 ± 0.5 | 0.11 ± 0.32 | 0 ± 0 | 0.02 ± 0.13 | 0.02 ± 0.13 | 0 ± 0 | 0 ± 0 | 1 ± 0 | 0.97 ± 0.17 | 0.72 ± 0.45 | 0.61 ± 0.49 | 0.81 ± 0.4 | 0.6 ± 0.49 | 0.27 ± 0.45 | 0.15 ± 0.36 | <0.001 | <0.001 | <0.001 | 0.01 (0.116, 0.193) |
| **Number of air entrance** | 1 ± 0 | 0.82 ± 0.39 | 0.4 ± 0.49 | 0.05 ± 0.22 | 0.08 ± 0.27 | 0.05 ± 0.22 | 0 ± 0 | 0 ± 0 | 1 ± 0 | 0.56 ± 0.5 | 0.11 ± 0.32 | 0 ± 0 | 0.02 ± 0.13 | 0.02 ± 0.13 | 0 ± 0 | 0 ± 0 | 1 ± 0 | 0.97 ± 0.17 | 0.72 ± 0.45 | 0.61 ± 0.49 | 0.81 ± 0.4 | 0.6 ± 0.49 | 0.27 ± 0.45 | 0.15 ± 0.36 | <0.001 | <0.001 | <0.001 | 0.01 (0.114, 0.195) |

Data expressed with mean ± standard deviation.

95%CI, 95% Confidence Interval; BROSJOD, Bronchiolitis Score of Sant Joan de Déu; ABSS, Acute Bronchiolitis Severity Scale.

^a^ Significant if p<0.05 (shown in red).

**Table S2** Qualitative outcome variables.

|  |  | **Assisted autogenic drainage group** | | | | | | | | **Slow expiration group** | | | | | | | | **Control group** | | | | | | | |  | |  |
| --- | --- | --- | --- | --- | --- | --- | --- | --- | --- | --- | --- | --- | --- | --- | --- | --- | --- | --- | --- | --- | --- | --- | --- | --- | --- | --- | --- | --- |
|  |  | **Baseline** | **10 minutes** | **20 minutes** | **1 hour** | **48 hours baseline** | **48 hours 10 minutes** | **48 hours 20 minutes** | **48 hours 1 hour** | **Baseline** | **10 minutes** | **20 minutes** | **1 hour** | **48 hours baseline** | **48 hours 10 minutes** | **48 hours 20 minutes** | **48 hours 1 hour** | **Baseline** | **10 minutes** | **20 minutes** | **1 hour** | **48 hours baseline** | **48 hours 10 minutes** | **48 hours 20 minutes** | **48 hours 1 hour** | **^a^p-value** | **Cramer´s V (95% CI)** | **Only mild vs. moderate levels** ^a^**p-value** |
| **ABSS score, n (%)** | Mild |  | 23 (37) | 58 (94) | 59 (95) | 59 (95) | 60 (97) | 62 (100) | 27 (44) |  | 37 (59) | 59 (94) | 47 (75) | 63 (100) | 63 (100) | 63 (100) | 2 (3) |  | 17 (25) | 54 (81) | 62 (93) | 49 (73) | 53 (79) | 60 (90) | 64 (96) | <0.001 | 0.57 (0, 1) | <0.001 |
|  | Moderate | 62 (100) | 39 (63) | 4 (6) | 0 (0) | 3 (5) | 2 (3) | 0 (0) | 0 (0) | 63 (100) | 26 (41) | 4 (6) | 0 (0) | 0 (0) | 0 (0) | 0 (0) | 0 (0) | 67 (100) | 50 (75) | 13 (19) | 5 (7) | 18 (27) | 14 (21) | 7 (10) | 3 (4) |  |  |  |
|  | Normal |  |  |  | 3 (5) |  |  |  | 35 (56) |  |  |  | 16 (25) |  |  |  | 61 (97) |  |  |  | 0 (0) |  |  |  | 0 (0) |  |  |  |
| **BROSJODD score, n (%)** | Mild |  | 33 (53) | 61 (98) | 62 (100) | 59 (95) | 60 (97) | 62 (100) | 61 (98) |  | 30 (48) | 60 (95) | 63 (100) | 63 (100) | 63 (100) | 63 (100) | 63 (100) |  | 11 (16) | 53 (79) | 63 (94) | 48 (72) | 52 (78) | 54 (81) | 62 (93) | <0.001 | 0.174 (0, 1) | <0.001 |
|  | Moderate | 62 (100) | 29 (47) | 1 (2) | 0 (0) | 3 (5) | 2 (3) | 0 (0) | 0 (0) | 63 (100) | 33 (52) | 3 (5) | 0 (0) | 0 (0) | 0 (0) | 0 (0) | 0 (0) | 67 (100) | 56 (84) | 14 (21) | 4 (6) | 19 (28) | 15 (22) | 13 (19) | 5 (7) |  |  |  |
|  | Normal |  |  |  |  |  |  |  | 1 (2) |  |  |  |  |  |  |  | 0 (0) |  |  |  |  |  |  |  | 0 (0) |  |  |  |

Data expressed with absolute and relative values (%).

95% CI, 95% Confidence Interval; BROSJOD, Bronchiolitis Score of Sant Joan de Déu; ABSS, Acute Bronchiolitis Severity Scale.

^a^ Significant if p < 0.05 (shown in red).

**Supplementary File Table 3** Quantitative outcome variables pairwise comparison p-values.

|  | **Baseline** | **10 minutes** | **20 minutes** | **1 hour** | **48 hours baseline** | **48 hours 10 minutes** | **48 hours 20 minutes** | **48 hours 1 hour** | **Average difference at 48 hours 1 hour (95% CI)** |
| --- | --- | --- | --- | --- | --- | --- | --- | --- | --- |
| **ABSS score** | | | | | | | | | |
| Assisted autogenic drainage group-Slow expiration group | 0.05 | 0.159 | 0.091 | <0.001 | <0.001 | 0.002 | 0.005 | <0.001 | 0 (0, 1) |
| Assisted autogenic drainage group-Control group | 0.4 | 0.177 | <0.001 | <0.001 | <0.001 | <0.001 | <0.001 | <0.001 | -2 (-2, -2) |
| Slow expiration group-Control group | >0.999 | <0.001 | <0.001 | <0.001 | <0.001 | <0.001 | <0.001 | <0.001 | -2 (-3, -2) |
| **BROSJOD score** | | | | | | | | | |
| Assisted autogenic drainage group-Slow expiration group | >0.999 | >0.999 | 0.001 | <0.001 | <0.001 | 0.004 | 0.007 | <0.001 | 0 (0, 1) |
| Assisted autogenic drainage group-Control group | >0.999 | <0.001 | <0.001 | <0.001 | <0.001 | <0.001 | <0.001 | <0.001 | -2 (-2, -2) |
| Slow expiration group-Control group | >0.999 | <0.001 | <0.001 | <0.001 | <0.001 | <0.001 | <0.001 | <0.001 | -2 (-3, -2) |
| **SpO_2_ (%)** | | | | | | | | | |
| Assisted autogenic drainage group-Slow expiration group | 0.116 | 0.009 | 0.008 | <0.001 | <0.001 | 0.001 | 0.003 | <0.001 | -1 (-1, 0) |
| Assisted autogenic drainage group-Control group | 0.352 | 0.103 | 0.642 | <0.001 | <0.001 | <0.001 | <0.001 | <0.001 | 1 (1, 1) |
| Slow expiration group-Control group | >0.999 | <0.001 | <0.001 | <0.001 | <0.001 | <0.001 | <0.001 | <0.001 | 2 (2, 2) |
| **Hearth rate (beats/min)** | | | | | | | | | |
| Assisted autogenic drainage group-Slow expiration group | <0.001 | <0.001 | <0.001 | 0.008 | 0.002 | 0.037 | 0.149 | 0.285 | -1 (-2, 0) |
| Assisted autogenic drainage group-Control group | 0.111 | 0.076 | <0.001 | <0.001 | <0.001 | <0.001 | <0.001 | <0.001 | -6 (-7, -4) |
| Slow expiration group-Control group | 0.1 | 0.002 | 0.024 | <0.001 | 0.016 | 0.003 | <0.001 | <0.001 | -5 (-6, -3) |
| **Respiratory rate (breath/min)** | | | | | | | | | |
| Assisted autogenic drainage group-Slow expiration group | 0.964 | 0.535 | 0.76 | 0.683 | 0.117 | 0.226 | 0.167 | 0.158 | 1 (0, 2) |
| Assisted autogenic drainage group-Control group | 0.043 | 0.143 | 0.286 | >0.999 | >0.999 | >0.999 | 0.735 | 0.001 | -3 (-4, -1) |
| Slow expiration group-Control group | 0.344 | 0.745 | >0.999 | 0.248 | 0.014 | 0.019 | 0.011 | <0.001 | -3 (-5, -2) |
| **Number of wheezing** | | | | | | | | | |
| Assisted autogenic drainage group-Slow expiration group | >0.999 | >0.999 | >0.999 | 0.467 | 0.467 | 0.467 | 0.467 | 0.467 | 0 (0, 0) |
| Assisted autogenic drainage group-Control group | <0.001 | 0.492 | 0.879 | >0.999 | 0.481 | >0.999 | >0.999 | >0.999 | 0 (0, 0) |
| Slow expiration group-Control group | 0.091 | >0.999 | 0.913 | 0.277 | 0.047 | 0.152 | 0.277 | 0.277 | 0 (0, 0) |
| **Retraction effort** | | | | | | | | | |
| Assisted autogenic drainage group-Slow expiration group | ^a^ | 0.467 | <0.001 | <0.001 | <0.001 | 0.001 | 0.24 | 0.467 | 0 (0, 0) |
| Assisted autogenic drainage group-Control group | ^a^ | >0.999 | 0.017 | <0.001 | <0.001 | <0.001 | <0.001 | <0.001 | -1 (-1, -1) |
| Slow expiration group-Control group | ^a^ | >0.999 | <0.001 | <0.001 | <0.001 | <0.001 | <0.001 | <0.001 | -1 (-1, -1) |
| **Number of crackling** | | | | | | | | | |
| Assisted autogenic drainage group-Slow expiration group | >0.999 | 0.704 | 0.055 | <0.001 | <0.001 | <0.001 | <0.001 | <0.001 | 0 (0, 0) |
| Assisted autogenic drainage group-Control group | 0.356 | 0.538 | <0.001 | 0.004 | 0.008 | 0.146 | 0.006 | <0.001 | -1 (-1, -1) |
| Slow expiration group-Control group | 0.343 | >0.999 | 0.096 | <0.001 | 0.016 | 0.001 | 0.01 | <0.001 | -1 (-1, -1) |
| **Inspiration-expiration rate** | | | | | | | | | |
| Assisted autogenic drainage group-Slow expiration group | ^a^ | 0.004 | 0.001 | 0.24 | 0.279 | 0.923 | ^a^ | ^a^ | ^a^ |
| Assisted autogenic drainage group-Control group | ^a^ | 0.017 | 0.001 | <0.001 | <0.001 | <0.001 | <0.001 | 0.003 | 0 (0, 0) |
| Slow expiration group-Control group | ^a^ | <0.001 | <0.001 | <0.001 | <0.001 | <0.001 | <0.001 | 0.003 | 0 (0, 0) |
| **Number of air entrance** | | | | | | | | | |
| Assisted autogenic drainage group-Slow expiration group | ^a^ | 0.004 | 0.001 | 0.24 | 0.279 | 0.923 | ^a^ | ^a^ | ^a^ |
| Assisted autogenic drainage group-Control group | ^a^ | 0.017 | 0.001 | <0.001 | <0.001 | <0.001 | <0.001 | 0.003 | 0 (0, 0) |
| Slow expiration group-Control group | ^a^ | <0.001 | <0.001 | <0.001 | <0.001 | <0.001 | <0.001 | 0.003 | 0 (0, 0) |
| **Number of wheezing with crackling** | | | | | | | | | |
| Assisted autogenic drainage group-Slow expiration group | <0.001 | 0.23 | 0.413 | 0.002 | 0.923 | 0.467 | 0.467 | <0.001 | 0 (0, 0) |
| Assisted autogenic drainage group-Control group | 0.01 | 0.552 | >0.999 | 0.002 | 0.286 | 0.88 | 0.88 | <0.001 | -1 (-1, -1) |
| Slow expiration group-Control group | 0.21 | 0.007 | >0.999 | <0.001 | 0.035 | 0.084 | 0.084 | <0.001 | -1 (-1, -1) |

^a^ Same values between groups with no differences.

95% CI, 95% Confidence Interval; BROSJOD, Bronchiolitis Score of Sant Joan de Déu; ABSS, Acute Bronchiolitis Severity Scale.

Significant if adjusted p < 0.05.

**Supplementary File Table 4** Qualitative outcome variables pairwise comparison p-values.

|  | **Assisted autogenic drainage group-Slow expiration group** | **Assisted autogenic drainage group-Control group** | **Slow expiration group-Control group** |
| --- | --- | --- | --- |
| **ABSS score baseline** | ^a^ | ^a^ | ^a^ |
| **ABSS Score 10 minutes** | 0.12 | >0.999 | <0.001 |
| **ABSS score 20 minutes** | >0.999 | 0.266 | 0.259 |
| **ABSS score 1 hour** | 0.012 | 0.098 | <0.001 |
| **ABSS Score 48 hours baseline** | 0.714 | 0.007 | <0.001 |
| **ABSS score 48 hours 10 minutes** | >0.999 | 0.021 | <0.001 |
| **ABSS score 48 hours 20 minutes** | ^a^ | 0.098 | 0.091 |
| **ABSS score 48 hours 1 hour** | <0.001 | <0.001 | <0.001 |
| **BROSJOD score baseline** | ^a^ | ^a^ | ^a^ |
| **BROSJOD score 10 minutes** | >0.999 | <0.001 | <0.001 |
| **BROSJOD score 20 minutes** | >0.999 | 0.007 | 0.056 |
| **BROSJOD score 1 hour** | ^a^ | 0.84 | 0.84 |
| **BROSJOD score 48 hours baseline** | 0.714 | <0.001 | <0.001 |
| **BROSJOD score 48 hours 10 minutes** | >0.999 | 0.007 | <0.001 |
| **BROSJOD score 48 hours 20 minutes** | ^a^ | <0.001 | <0.001 |
| **BROSJOD score 48 hours 1 hour** | >0.999 | 0.413 | 0.406 |

^a^ Unique level without differences in both groups.

BROSJOD, Bronchiolitis Score of Sant Joan de Déu; ABSS, Acute Bronchiolitis Severity Scale.

Significant if adjusted p < 0.05.

**Post hoc tests and significant differences for the group × time interaction of qualitative variables**

Post hoc tests have shown no baseline differences between groups. In the measurement 1-hour after the baseline measurement, in the ABSS, significant differences were observed in the AAD group, where all subjects went from moderate severity at baseline to normal level (n = 3) and mild severity (n = 59). Significant final differences one hour after treatment at the 48-hours session were observed between all groups in the ABSS, with an increase in the proportion of patients with a normal level in the PSE group and less in the AAD group, a decrease in patients with a moderate level in all groups, especially in the AAD group, while patients with a mild level increased in the Control group and decreased in the PSE group. In the BROSJOD the final differences appear between the AAD group and Control group, and PSE group and Control group groups 20-minutes after treatment at the 48 hours session, with a greater reduction of patients with a higher-level moderate in both the AAD group and the PSE group versus the Control Group (Supplementary File Table 2 and Table 4).

For the qualitative variables, significant differences for group × time interaction was demonstrated in both ABSS (M2(4) = 218.845, p < 0.001, Wolf test p-value = 0.973) and BROSJOD (M2(2) = 120.589, p < 0.001, Wolf test p-value = 0.818) with a large effect size in the first and small in the second, although significant in both cases (Supplementary File Table 3).

**Figure S1** Heart rate trends over time in AAD, PSE, and control groups (mean ± SD per time point).


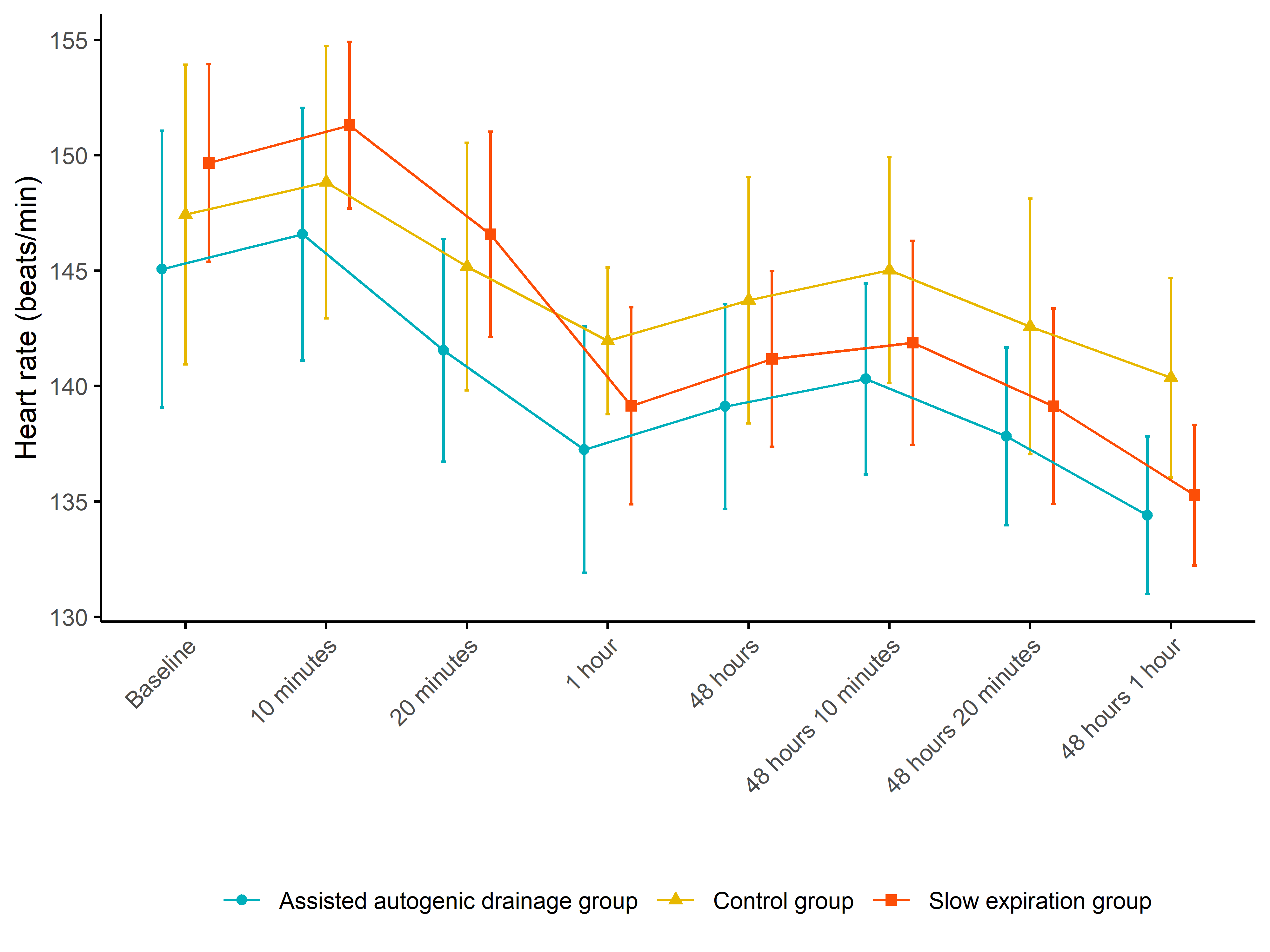

Supplement: Supplementary file 1 [file mmc1.docx]
